# Untreated depression among persons living with human immunodeficiency virus in Kazakhstan: A cross-sectional study

**DOI:** 10.1371/journal.pone.0193976

**Published:** 2018-03-28

**Authors:** Dina Terloyeva, Zhamilya Nugmanova, Gulzhakhan Akhmetova, Aikan Akanov, Nimish Patel, Victoria Lazariu, Lisa Norelli, Louise-Anne McNutt

**Affiliations:** 1 Department of HIV infection and Infection Control, Asfendiyarov Kazakh National Medical University (KNMU), Almaty, Kazakhstan; 2 Department of Epidemiology and Biostatistics, Drexel University, Philadelphia, Pennsylvania, United States of America; 3 Almaty City AIDS Centre, Almaty, Kazakhstan; 4 Department of Public Health, Asfendiyarov Kazakh National Medical University (KNMU), Almaty, Kazakhstan; 5 Albany College of Pharmacy & Health Sciences, Albany, New York, United States of America; 6 Department of Epidemiology and Biostatistics, University at Albany, State University of New York, Rensselaer, New York, United States of America; 7 Department of Psychiatry, Albany Medical College, Albany, New York, United States of America; 8 Institute for Health and the Environment, University at Albany, State University of New York, Rensselaer, New York, United States of America; The University of New South Wales, Neuroscience Research Australia, AUSTRALIA

## Abstract

**Background:**

In Kazakhstan, scarce official prevalence data exists for mood disorders. This study investigates the occurrence of depressive symptoms among people living with HIV/AIDS (PLWHA), and the relationship between depressive symptoms, HIV treatment initiation and antiretroviral treatment (ART) adherence.

**Methods:**

A cross-sectional study was conducted among patients seen at the Almaty AIDS Center between April and December 2013. Two data sources were used: 1) self-administered survey that included the Patient Health Questionnaire (PHQ-9) to capture depression symptoms and 2) medical record review. Two primary outcomes were evaluated with log-binomial models and Fisher’s exact test: the relationship between depression symptoms and 1) HIV treatment group, and 2) HIV adherence.

**Results:**

Of the 564 participants, 9.9% reported symptoms consistent with a depressive disorder. None had received treatment for depression. Among those not on ART, a relationship between depressive symptoms and low CD4 counts (≤ 350 cells/mm^3^) was evident (7.1% for CD4 ≤ 350 cells/mm^3^ vs. 0.9% for CD4 > 350 cells/mm^3^, p = 0.029). In multivariable analysis, a higher prevalence of depressive symptoms was statistically associated with ART treatment, positive hepatitis C virus (HCV) status, and being unmarried. For those taking ART, treatment adherence was not statistically associated with a lower prevalence of depressive symptoms (12.5% vs 20.0%, p = 0.176); limited power may have impacted statistical significance.

**Conclusions:**

Untreated depression was found among PLWHA suggesting the need to evaluate access to psychiatric treatment. A collaborative strategy may be helpful to optimize HIV treatment outcomes.

## Introduction

### HIV trends and current status in Eastern Europe and Central Asia

In Eastern Europe and Central Asia, the incidence of HIV is increasing at an alarming pace while elsewhere it is either declining or at stasis [1]. According to the Joint United Nations Program on HIV/AIDS (UNAIDS), a 57% average annual increase of new adult infections has occurred between 2010 and 2015 in the United Nations-designated Eastern Europe and Central Asia region [1]. At a time when the AIDS-related death rate is decreasing globally, largely due to effective antiretroviral therapies (ART), this region is experiencing a concomitant increase in AIDS-related deaths [1]. Nearly all of these new HIV infections and deaths have been among those living in the former Soviet Union and its historically allied countries, a region comprising a population of approximately 330 million people [1]. Given the disparate trends in HIV globally, this region is in exigent need of a robust analysis to identify barriers to HIV prevention and treatment which will, in turn, inform the development and implementation of effective interventions.

Kazakhstan, a former Soviet Republic with a population of 18 million people [2], provides a useful environment in which to study barriers that impede successful HIV treatment and prevention in the region, and to some extent, more broadly. Kazakhstan with 17% increase, and its largest city (Almaty) with 20% increase in registered new adult HIV infections from 2010 to 2015, reflect the HIV epidemic rates in the region; actual increases likely are greater. AIDS deaths have also risen during this time period [3].

The economic collapse and diaspora caused by the fall of the Soviet Union resulted in a massive increase in illicit drug using behavior, and incipient HIV transmission among persons who inject drugs (PWID) [4, 5]. Although the epidemic was initially fueled by illicit drug use, transmission soon bridged to partners of PWID through heterosexual transmission. Sexual transmission became the leading transmission route in 2010; in 2014 59.8% of new cases were reportedly due to sexual transmission (mostly heterosexual), while 31.7% of new cases were classified as parenteral transmission [6, 7]. The HIV epidemic in Kazakhstan, similar to the region, is considered to be concentrated in high-risk population groups: PWID, commercial sex workers (CSW), men who have sex with men (MSM), and prisoners [7]. According to the national active surveillance system, the prevalence of HIV among PWID was 8.2%, while among CSW it was 1.3%, 3.2% among MSM, and 3.9% among prisoners [7].

There are 16 treatment centers specializing in HIV strategically located across Kazakhstan [8]. The Almaty AIDS Center serves about 2660 PLWHA and provides prevention and screening services to the city of 1.7 million and its surrounding areas. The AIDS centers provide free HIV testing, counseling and treatment. Kazakh HIV treatment guidelines are consistent with the World Health Organization (WHO) guidelines, however, implementation of changes tend to be delayed by approximately a year due to the need for regulatory modifications and approval. Currently, individuals with a newly diagnosed HIV infection are immediately offered free ART treatment. At the time of our study, Kazakh treatment guidelines recommended treatment when CD4 count was ≤ 350 cells/mm^3^ at clinical stage I or II; all patients were eligible if classified as clinical stage III or IV, irrespective of the CD4 count [9].

### Depression diagnosis and treatment in Kazakhstan

The organization of medical care delivery in Kazakhstan, like most former Soviet Republics, is partitioned. Medical licenses are provided for a specific specialty and boundaries are enforced. Medical centers, called polyclinics, might have multiple medical specialties in the same building, but integrated care is scarce and virtually none offer mental health services. Yet, it is well established that mood disorders can exacerbate comorbid chronic conditions, such as asthma, diabetes, arthritis, angina and pain [10, 11]; they are associated with HIV [12–14], and can affect compliance to treatment [15], including potentially diminishing adherence to ART [16–19].

Depression can be officially diagnosed and treated only within psychiatric facilities by trained psychiatrists. However, psychiatric facilities are predominantly utilized by patients with severe mental health disorders, typically presenting with psychotic symptoms. Patients with depressive symptoms generally do not seek service and are not referred by physicians unless the symptoms are extraordinarily severe. In addition to the stigma associated with obtaining psychiatric services, the Soviet Union’s political abuse of psychiatry for non-medical purposes continues to inhibit some from seeking services [20]. Unfortunately, the compartmentalized mental health care model and longstanding severe aversion to using psychiatric services introduces important barriers to integrated mental health diagnosis and treatment into general medical care [21].

Little population-based epidemiological data exists to estimate incidence and prevalence of depression in Kazakhstan. A 2001 population-based survey conducted in Kazakhstan estimated 3.8% of adults had high psychological distress scores, a measure that includes symptoms of depression, anxiety and stress [22]. More recently, the prevalence of registered cases of International Statistical Classification of Diseases and Related Health Problems, 10^th^ revision (ICD-10) mood disorders per 100,000 population was 22.5 in 2010 and 21.0 in 2011 [23], representing approximately 0.02% of the adult population. This is a vast underestimate: the worldwide prevalence of mental disorders (general category) is estimated to be about 5% [24], and the U.S. National Institute of Mental Health estimates, based on a population survey, that 9.5% of all adult Americans have a mood disorder [25, 26]. The official Kazakh prevalence is an underestimate for a variety of reasons, but chief among them are the lack of population studies to measure depression specifically, limited access to diagnosis and treatment, and the aforementioned social barriers.

While no Kazakh data specific to PLWHA exists to estimate depression prevalence, two studies provide insight. A preliminary study was conducted using qualitative methods to investigate the mental status of, and access to, mental health services for PWID who are living with HIV [27]. Focus groups of PWID discussed the mental health problems in the community and semi-structured interviews with a non-governmental organization (NGO) and medical staff, as well as other key stakeholders were conducted. These studies suggest that access to mental health care for PLWHA is extremely limited for this vulnerable population [27]. Another study administered the Brief Symptom Inventory 18-item to 728 PWID; approximately 25% of participants were infected with HIV. Depression prevalence was not estimated. The study did not find an association between HIV infection and depressive symptoms, however there were no data on treatment factors related to HIV [28].

### Purpose of the study

In Kazakhstan, HIV physicians are interested in improving patient outcomes, especially patient willingness to initiate ART and adherence to the prescribed medication regimen once started. There is some evidence that depression is associated with poor ART adherence [29–34], and we hypothesize that depression would influence initiation and adherence to medication in Kazakhstan. This study investigated depression symptoms among PLWHA seen at a treatment center to ascertain the proportion of patients who may be eligible for depression treatment. The objective was to understand if the challenges in HIV treatment utilization, such as delay in treatment initiation, discontinuation of treatment, and suboptimal treatment adherence were partially due to undiagnosed and untreated depression. In addition, we attempted to assess sex for effect modification guided by the existing literature that shows sex differences in the prevalence, onset, and manifestation of depression, and response to antidepressant treatment [35–38]. Given the expanding HIV epidemic in this region, it is exigent to improve HIV treatment initiation and adherence in Kazakhstan by identifying levers which promote population-based treatment strategies, such as improving collaborative care for HIV.

## Methods

A cross-sectional study was conducted among patients of the Almaty AIDS Center during the study period (April–December 2013). Individuals were eligible for the study if they were: 1) age ≥ 18 years, 2) had documentation of laboratory-confirmed HIV infection, 3) spoke Kazakh or Russian, and 4) were not intoxicated at the time of their clinic visit. Patients recently (≤ 3 months) linked to care at the Almaty AIDS Center were not evaluated for inclusion in the study. Eligible patients were provided written and oral informed consent; consenting patients were enrolled. The study was approved by the Research Ethics Committee at the Kazakh National Medical University (KazNMU), registered with US HHS OHRP (Number: IRB00003734 - Kazakh Natl Med U-Kazakhstan Ethical Committee-IRB #1). A small gift (telephone calling cards worth approximately four USD) was provided to participants.

Two sources of data were used for these analyses: 1) self-administered questionnaire completed by patients, and 2) medical record review. Pertinent data elements on the questionnaire included demographics and clinical characteristics including symptoms of depression. Diagnostic and treatment information related to HIV and depression were abstracted from medical records.

### Depression measures

Symptoms of depression were assessed with responses to the Patient Health Questionnaire-9 (PHQ-9) [39] in its official Russian translation [40]. The nine items on the PHQ-9 directly correspond to the nine *Diagnostic and Statistical Manual of Mental Disorders IV (DSM-IV)* criteria for depressive disorders. The items ascertain how frequently the individual has experienced each diagnostic criterion during the last two weeks and assigns a score of 0, 1, 2 and 3 for not at all, several days, more than half of the days, and nearly every day, respectively. To assess the severity of depressive symptoms, item scores were summed across all symptoms and then categorized as follows: mild if the resulting summative score was 5–9, moderate for sums of 10–14, moderately severe for sums 15–19, and severe for sums of 20–27 [40].

A participant was classified as having symptoms consistent with a *major depressive disorder* if he or she reported having experienced five or more symptoms for the past two weeks at least “more than half of the days,” with at least one of the reported symptoms consistent with anhedonia, or feeling depressed. For those reporting two to four symptoms, including anhedonia or depressed mood, at least “more than half of the days” or “nearly every day” for the past two weeks, the symptoms were classified as consistent with *other depressive disorder*. For the binary depression prevalence measure, participants were coded as having depression if their responses met the criteria for either major depressive disorder or other depressive disorder [40].

Substantial measurement assessment has been conducted on the original English language PHQ-9 in US primary care populations; it is a validated questionnaire to measure mood disorders [39, 41]. The PHQ-9 has also been validated in multiple other languages [42–53]. A measurement invariance study conducted among PLWHA in the US found some differential item functioning with respect to race (white, African-American), sex, and age occurred, however the impact in the diagnostic range of the summary score for depression was minimal [54]. Face validity of the Russian translation of the PHQ-9 was determined by a Kazakh psychiatrist and a clinician, both native Russian speakers. Content validity was established in English [39] and confirmed in the Russian version by a Kazakh psychiatrist. Construct validity of the Russian version was demonstrated by the strong association between the PHQ-9 and general health (Table 1); internal consistency was assessed using Cronbach’s alpha (α = 0.95).

**Table 1:** Factors associated with being classified as having depressive symptoms in HIV-infected individuals.

Overall	N	% Depression	Prevalence Ratio	95% CI	p-value*
**Sex**					0.17
Male	311	8.4%	Referent		
Female	253	11.9%	1.4	0.9–2.3	
Missing	0				
**Age**					0.39
18–34	236	8.1%	Referent		
35–44	218	11.9%	1.5	0.8–2.6	
45 and older	101	10.9%	1.4	0.7–2.7	
Missing	9				
**Ethnicity**					0.42
Kazakh	161	6.8%	Referent		
Russian	298	10.4%	1.5	0.8–2.9	
Other	95	10.5%	1.5	0.7–3.5	
Missing	10				
**Education**					0.16
College/University	73	5.5%	Referent		
Secondary school and below	483	10.8%	2.0	0.7–5.3	
Missing	8				
**Marital Status**					0.001
Married/Cohabitate	395	6.8%	Referent		
Single	104	17.3%	2.5	1.5–4.4	
Previously married**	62	17.7%	2.6	1.4–5.0	
Missing	3				
**Self-assessed health**					<0.001
Good/very good	333	2.7%	Referent		
Neither good nor bad	186	14%	5.2	2.5–10.8	
Bad/very bad	34	52.9%	19.6	9.6–40.2	
Missing	11				
**History of injection drug use**					0.06
No	262	7.3%	Referent		
Yes	262	12.2%	1.7	1.0–2.9	
Missing	40				
**Years with HIV**					0.78
1 year or less	138	8.7%	Referent		
2–4 years	187	9.6%	1.1	0.6–2.2	
5+years	239	10.9%	1.3	0.7–2.4	
Missing	0				
**HCV status**					0.01
No	301	7.0%	Referent		
Yes	263	13.3%	1.9	1.1–3.2	
Missing	0				
**No ART**					0.01
>350 CD4	107	0.9%	Referent	0.9–62.3	
≤350 CD4	84	7.1%	7.6	1.6–115.5	
**Former ART**	32	12.5	13.4		
**ART**					
>350 CD4	102	13.7%	14.7	2.0–109.7	
≤350 CD4	220	12.7%	13.6	1.9–98.8	
Missing	19				

* Fisher’s Exact Test or generalized hypergeometric test if there were cells with expected counts less than 5, otherwise–chi-square test.

**Category “Previously married” includes those divorced, separated or widowed.

### HIV-related measures

Medical records were reviewed for information on treatment history and comorbidities, including whether the patient was taking ART or obtaining any form of treatment for depression (e.g., medication, counselling). Year of HIV diagnosis was abstracted. Blood tests for CD4 cells/mm^3^ were conducted monthly and recorded as part of the routine medical care; the CD4 count obtained from the blood test conducted on the day of survey was abstracted. In Kazakhstan, a patient was eligible (at the time of the study) for ART if his/her CD4 values was ≤ 350 cells/mm^3^ at clinical stage I or II; all patients were eligible if classified as clinical stage III or IV, irrespective of the CD4 count. Additionally, three-month pill counts for those on ART were documented as a measure of treatment adherence. Patients were classified as “adherent” if at least 90% of pills were missing and reported consumed.

### Demographic characteristics and potential confounders

Demographic characteristics collected on the self-administered questionnaire to describe the study population and assess as possible confounding and effect modifying included age, sex, ethnicity (Kazakh, Russian, and other), marital status (single, married/cohabitating, previously married), and education (college/university, and secondary school or below). Additional factors collected to determine potential confounding were likely mode of infection (eg, history of injecting drug use, and other; self-reported), and HCV co-infection (recorded in medical record).

### Statistical analyses

Descriptive analyses were conducted using frequencies and percentages for categorical variables and means with standard deviations for continuous variables. Categorization of continuous variables (e.g., age) was conducted to maximize information while minimizing potential of residual confounding [55, 56] Point biserial correlations were computed on dichotomized factors to display relationships among factors. Missing data patterns were assessed to determine if an association with depression existed.

To estimate the relationship between HIV treatment and prevalence of depression, patients were classified based on treatment status (taking ART, formerly taking ART, ART not initiated) and further classified by CD4 count ≤ 350 cells/mm^3^ (yes, no). PLWHA who have not initiated ART and who had CD4 count > 350 cells/mm^3^ were not eligible for treatment at the time; those with CD4 count ≤ 350 cells/mm^3^ were eligible but had not initiated treatment. PLWHA taking ART and had a CD4 count ≤ 350 cells/mm^3^ were defined as having disease control (viral load was not routinely measured at the time). We hypothesized that patients eligible for ART (CD4 count ≤350 cells/mm^3^) with no history of taking ART are not initiating HIV treatment partially due to depressive symptoms and hence would have higher prevalence of depression compared to those who are not yet eligible for ART. We further stratified patients by sex because we expected the prevalence of depression would be higher among female participants compared to male participants, because standard depression scales tend to be more sensitive in females compared to males [36]. Fisher’s exact tests, or the generalized hypergeometric tests, were used to test the association. Log binomial models with robust errors were used to assess potential confounders, including sex, age, marital status, hepatitis C status, history of injecting drugs and years with HIV diagnosis. The final model was adjusted for a sufficient subset of confounders [55]. Using a backward selection approach, we dropped variables from the model which had a p-value greater than 0.05. We then investigated if any variable dropped from the model was a confounder for any of the remaining variables by adding back the variable and assessing if the parameter estimates changed substantively [55]. Interaction terms were added to assess effect modification of treatment categories, none were statistically significant (p>0.05 for each).

A sequential regression with a multiple imputation approach was used to impute missing values [57, 58]. This Bayesian approach, which uses all the variables in the analyses and assumes data is missing at random, preserves the existing correlations among covariates and the interdependencies in the data. A total of five completed datasets were generated and the results from the regression analyses were pooled across the imputed datasets. Missing data imputations were performed with IVEware software, and all other analyses were performed using SAS® version 9.4 (SAS Institute, Cary, NC). Further, we conducted the same analysis including only participants with complete data to identify if any substantial shifts in parameter estimates occurred.

Model fit was assessed through a residual analysis within covariate patterns. Within each covariate pattern residuals were calculated as the difference between the average prediction and the observed proportion of depressive symptoms. The distribution of residuals obtained was weighted by the number of people within the covariate pattern. A t-test failed to reject the null hypothesis that the mean is equal to zero (p-value >0.429).

To assess the relationship between depression and adherence to ART treatment, Fisher’s exact test was used to test the hypothesis among treated patients. Further, log binomial regression analysis was conducted; adjusting for sex, age, marital status, hepatitis C status, history of injecting drugs and years with HIV diagnosis; to determine if confounding by demographic or clinical factors occurred.

## Results

Of the 600 surveys, all conducted in Russian, 36 were found to be duplicates, thus 564 (94%) surveys were included in these analyses. Data were complete for the majority of factors; self-reported injecting drug use had the largest number of missing values (n = 40, 7%). No statistically significant association was identified between missing data distributions and depression (see S2 Table in Supporting Information for missing data summary).

The mean age of study participants was 38.0 years (standard deviation = 11.5 years) and about half were male (55.1%). About half reported Russian (53.8%) ethnicity; about a quarter reported Kazakh (29.1%) ethnicity. Most patients reported completing high school (83.5%) and reported being in good health (58.5%). About half indicated the mode of HIV transmission was sexual (51.6%) while 42.0% reported parenteral/intravenous drug use. Less than five participants reported MSM as a risk factor for transmission. The median (interquartile range, IQR) duration of HIV infection was 4 (2–6) years. Current antiretroviral therapy was reported by 59.0% of PLWHA; overall 59.7% of participants had CD4 values below 350 cells/mm^3^.

Point biserial correlations (S1 Table) of covariates show the strongest correlation between self-reported PWID and HCV infection (0.440, p<0.01). Sex was significantly associated with many factors; men were more likely to be PWID, HCV infected, older, living with a HIV diagnosis longer and have CD4 count below 350 cells/mm^3^ (S1 Table). Among men 67.3% (200/297) reported a history of injecting drugs, while only 27.3% (62/227) of women identified themselves as PWID (p<0.0001). Russian ethnicity was positively associated with history of injecting drugs, HCV status, higher education, and negatively associated with being married. People whose self-assessed health below good or very good were more likely to have longer HIV infection, CD4 cell counts below 350 cells/mm^3^, HCV, and were less likely to me married (S1 Table).

The proportion of participants who met the PHQ-9 criteria for a depressive disorder was 9.9%. No patients were treated for depression nor had a diagnosis of depression recorded in their medical record.

Table 1 presents bivariate analyses of demographic and clinical characteristics for those who did and did not meet the criteria for a depressive disorder. Depression prevalence increased as age increased. There was no association between education and depression nor between ethnicity and depression. Participants who were married were less likely to report symptoms consistent with a depressive disorder. Although female patients were more likely to report depression, sex differences were not significant. Education was not significantly associated with depression. People reporting depression were more likely to assess their health status as “neither good nor bad”, or “bad/very bad”. The association between self-reported history of injection drug use and symptoms of depressive disorder was marginally significant (p = 0.055). Patients infected with hepatitis C were significantly more likely to have symptoms of depressive disorder compared to those not infected (p = 0.012). There was no statistically significant association between years since HIV diagnosis and depressive symptoms.

Table 2 summarizes detailed information on responses to the depression items. While not statistically significant, across all the items, women reported more depression symptoms, more often met the criteria for depressive disorders (major and other) and reported higher severity for each symptom compared to men.

**Table 2:** Classification of depressive symptoms and severity by sex.

	Males	Females	
	% (n)	% (n)	
Classification of symptoms by depression scale	N = 311	N = 253	p-value*
Major	4.2 (13)	8.7 (22)	0.08
Other	4.2 (13)	3.2 (8)	
Symptom severity			
Classification of depressive symptoms			
Mild depression	45.6 (141)	39.5 (100)	0.17
Moderate depression	4.9 (15)	5.9 (15)	
Moderately severe depression	3.9 (12)	5.1 (13)	
Severe depression	0	1.2 (3)	

* Fisher’s exact test assessed the association between sex and levels of depressive symptoms

Table 3 presents results of current HIV treatment status, sex and depressive symptoms. Participants were categorized into five groups based on a combination of use of ART treatment (never, former, current) and CD4 cell count. Among patients *not* taking ART, those with a low CD4 cell count (≤ 350 cells/mm^3^) reported more depressive disorders than those with a higher CD4 cell count (7.1% vs. 0.9%, p = 0.029).

**Table 3:** Distribution of depressive symptoms in males and females, by cd4 categories, multivariable analysis.

Proportion of patients with depressive symptoms	Patients not on ART			Former ART patients	Patients on ART		
	CD 4 cell count				CD 4 cell count		
	>350 cells/mm^3^	≤ 350 cells/mm^3^			>350 cells/mm^3^	≤ 350 cells/mm^3^	
	% (n)	% (n)	p-value		% (n)	% (n)	p-value
Males	0% (0/50)	4.3% (2/47)	0.23	16.7% (2/12)	10.2% (5/49)	10.6% (15/142)	0.59
Females	1.8% (1/57)	10.8% (4/37)	0.08	10% (2/20)	17.0% (9/53)	16.7% (13/78)	0.57
Overall	0.9% (1/107)	7.1% (6/84)	0.03	12.5% (4/32)	13.7% (14/102)	12.7% (28/220)	0.47
Years since HIV diagnosis							
Mean (SD)	3.1 (2.7)	4.2(2.6)	0.01	3.1 (2.5)	4.9 (3.3)	4.4 (3.2)	0.17

For those *taking* ART, the sex difference persisted (females were more likely to report depressive symptoms than males albeit not statistically significant) but the CD4 count relationship did not persist. In other words, the frequency of patients reporting depressive symptoms was similar across both CD4 count strata, and higher than those not yet eligible for ART (13.0% vs. 0.9%, p<0.01). Patients who chose to stop ART had similar levels of depressive symptoms as those taking ART. The regression modelling showed minimal confounding occurred by age, sex, education, and marital status. Analysis stratified by sex found no interactions, rather females reported more depressive disorder symptoms than males across the ART and CD4 cell count categories. Although prevalence of depressive symptoms was higher among those taking Efavirenz compared to those taking other antiretrovirals (14.3% vs 10.9%, respectively), this relationship was not statistically significant (p = 0.372).

Of the 564 study participants, about 10% had missing data, thus a multiple imputation method was used to impute values for regression analyses. In the multivariable regression analyses sex, age, time since HIV diagnosis and history of injection drug use did not reach statistical significance and were not in the sufficient subset of confounders utilized, therefore were not included in the final model. Higher prevalence of depression was associated with current or previously received ART treatment or having a CD4 count < 350 cells/mm3 compared to never using ARTs and having a CD4 count > 350 cells/mm3 (Table 4). Among patients not taking ART, the prevalence of depression was higher for those with CD4 counts ≤ 350 cells/mm^3^ compared to those with higher CD4 counts; the confidence limits and p-value for this result do not agree due to the approximation used to pool the estimates across the imputed datasets.

**Table 4:** Factors associated with being classified as having depressive symptoms in hiv-infected individuals*.

**Overall**	N**	**% Depression**	Adjusted Prevalence Ratio**	**95% CI**	**p-value**
**No ART**					
>350 CD4	107	0.9%	Referent		
≤350 CD4	84	7.1%	5.8	0.8–40.3	0.07
**Former ART**	32	12.5	9.9	1.5–66.1	0.02
**ART**					
>350 CD4	102	13.7%	8.6	1.3–54.9	0.02
≤350 CD4	220	12.7%	9.1	1.1–77.8	0.04
Missing	19				
**HCV status**					
No	301	7.0%	Referent		
Yes	263	13.3%	1.7	1.0–2.8	0.04
Missing	0				
**Marital Status**					
Married/Cohabitate	395	6.8%	Referent		
Single	104	17.3%	2.4	1.4–4.3	0.002
Previously married***	62	17.7%	2.3	1.2–4.3	0.01
Missing	3				

*Sex, age, ethnicity, education, history of injection drug use, years living with HIV were not included in the model because they were not statistically significant (p>0.05) nor in the sufficient confounder set for variables remaining in the model.

**Pooled results from five complete datasets generated using a sequential regression multiple imputation technique.

***Category “Previously married” includes those divorced, separated or widowed.

There were 333 patients (59%) receiving ART medications. Of these patients, 267 (80.2%) were on a non-nucleoside reverse transcriptase inhibitor (NNRTI)-based regimen and 28 (8.4%) were on a protease inhibitor (PI)-based regimen. The remainder of patients were receiving non-traditional ART regimens composed of only nucleoside reverse transcriptase inhibitors (NRTIs). The distribution of NNRTIs used among the 267 NNRTI-based ART regimen recipients were efavirenz (n = 196, 73.4%) and nevirapine (n = 71, 26.6%). Recipients of ART were significantly older (38.6 ± 9.2 versus 35.1 ± 8.2 years, p < 0.001) and more likely to be male (59.2% vs 49.4%, p = 0.02). There were no significant differences between ART and non-ART users with respect to ethnicity (p = 0.55), education (p = 0.98), marital status (p = 0.35), HCV status (p = 0.07), and history of injecting drugs (p = 0.07).

A relationship between poor adherence (as measured by pill counts) and depression did not reach statistical significance, however the proportion of symptoms consistent with a depressive disorder was higher among those with poor adherence than among those who were adherent (20.0% vs. 12.5%, p = 0.172) (Table 5).

**Table 5:** Distribution of depressive symptoms in males and females, by adherence categories.

	Adherence		
	≥ 90%*	<90%	
	% (n)	% (n)	p-value
Males	7.2% (5/69)	13.6% (3/22)	0.296
Females	18.3% (11/60)	27.8% (5/18)	0.287
Overall	12.4% (16/129)	20.0% (8/40)	0.172

* **≥**90% adherence means that at least 90% of pills were consumed during the last three months.

## Discussion

Approximately 10% of persons living with HIV seen at the Almaty AIDS Center reported symptoms consistent with an acute depressive episode; no patient was treated for depression. The estimated prevalence falls in the middle of prevalences estimated by a WHO study of PLWHA that utilized clinical diagnostic interviews in five countries on four continents (range: 0% to 18.4% with an average of 7.1%), [12, 14] and in the lower half of prevalence estimates when compared to studies of depression among PLWHA conducted worldwide (range 3% to 63%) [59, 60]. The PHQ-9 tends to result in lower prevalence estimates [59]. However, many factors impact estimation of depression prevalence among PLWHA, including measurement instrument, sample characteristics and culture. It is clear from multiple studies that PLWHA are more likely to report depressive symptoms compared to those not infected with HIV [14].

Although depression is a serious concern for this HIV-positive population, there was no history of diagnosis, referral to a psychiatrist or treatment. Mental health care is free of charge in Kazakhstan yet structural barriers limit access to diagnosis and treatment to specialized psychiatric clinics. While patients typically are not seen by psychiatric specialists unless the depressive disorder is extremely severe or accompanied by a psychotic episode; various antidepressants in multiple classes, including affordable ones, are available in Kazakhstan. Also, psychologists are available in some AIDS centers as well as in primary and secondary health care networks, but it is unclear whether they are trained in evidence-based diagnosis and treatment strategies. Unfortunately, this lack of coordinated care has not been addressed despite the publication of qualitative research findings in 2007 [27]. If diagnosis and treatment for depression were integrated within the AIDS Centers, patients might be more accepting of treatment because they have established relationships with providers. Investigation of an integrated model with psychiatrists seeing patients at AIDS Centres is needed to determine if such a model can work in Kazakhstan. While such innovations require an initial investment in training, treatment for depression is not costly relative to ART. Thus, initial programmatic costs might be offset by improved adherence to HIV treatment. Given the limited availability of psychiatrists, a prudent longer term strategy would be to expand depression screening and treatment to primary care. The situation in Kazakhstan is representative of the region, thus providing an opportunity to develop strategies that could be exported to neighbouring countries. With an expanding economy and increased regional influence, Kazakhstan is in a good position to take a lead in this initiative.

Among patients who had not initiated ART, depressive symptoms were more common among those eligible for ART treatment. That is, we found a relationship between CD4 counts ≤ 350 cells/mm^3^ and depressive symptoms among those *not* taking ART. This is consistent with other research which found an association between depression and CD4 cell levels [16, 61–64]. However, it is difficult to parse the temporal relationship between CD4 cell counts, ART treatment and depression. While it follows that untreated depression could affect a patient’s choice not to initiate ART, which would, in turn, lead to suppressed CD4 cell levels, the research is somewhat mixed. Tegger, et al., found that depression made HIV-positive patients less likely to start ART [65], but Goodness, et al., did not find an association between depression and ART initiation in a population of alcohol abusers [66]. However, Goodness, et al., *did* find that depression accounted for delayed initiation of ART, and Tegger, et al., found that treating depression could increase the likelihood of ART acceptance [65]. Among patients who had initiated ARTs, depression symptoms were not associated with CD4 count; patients with low and high CD4 counts, including those who have discontinued treatment, have comparatively high levels of depression. Our hypothesis for this finding is that PLWHA who initiate ART are sicker. Further study is needed to determine if this is the case.

While the patients in our study who were adhering to ART treatment reported fewer depressive symptoms than those with poor adherence, the association did not reach statistical significance. Larger studies are needed to develop more precise estimates of depression and ART adherence. There is, however, consistency between our findings and previous studies regarding the negative effects of depression on ART adherence [16, 29, 30, 33, 67, 68]. Additionally, our results are consistent with prior research that history of injecting drug use was not associated with ART adherence [69, 70]. Further, previous research strongly suggests that antidepressant treatment can increase patients’ adherence to ART [18]. Thus, full clinical assessment, including mental health assessment, may be useful for PLWHA. The importance of starting and adhering to ART treatment cannot be overstated in terms of survival, especially for a vulnerable population already demonstrating a tendency toward risk behaviours, such as injection drug use [71].

It is important to emphasize that integrating depression and HIV treatment requires thoughtful consultation. The initiation of ART in the context of concomitant treatment for depression is complex due to overlapping metabolic pathways which can cause or exacerbate depressive symptoms during treatment. Specifically, a small proportion of patients using efavirenz—the WHO first-line non-nucleoside reverse transcriptase inhibitor (NNRTI) [72]—may experience severe depression (approximately 2%), suicidal ideations (<1%) and non-fatal suicide attempts (<1%) [20]. Nevirapine is an alternative NNRTI with interactions involving mirtazapine, escitalopram and venlafaxine. When NNRTIs are not appropriate, protease inhibitors are an alternative. Lopinavir/ritonavir is the most commonly used protease inhibitor in Kazakhstan. Patients using ART regimens that include the use of lopinavir/ritonavir should avoid concomitant use of several antidepressants like escitalopram, fluoxetine, paroxetine, mirtazapine, sertraline and venlafaxine because of the risk of tachyarrhythmia (specifically, QTc interval prolongation). Additionally, the use of fluvoxamine, as well as lopinavir/ritonavir (all are substrates of either CYP2D6 or CYP3A4), should be avoided because they interfere with drug metabolism placing patients at risk for anti-depressant-related toxicities [73]. Collectively, these issues underscore a universal need to expand access to newer ART agents with fewer drug-drug interactions involving antidepressants.

### Limitations

Limited resources resulted in a relatively short questionnaire and medical record review for this cross-sectional study. Education level was obtained, but not further information on socioeconomic status level. Additionally, alcohol abuse and non-injecting drug abuse were not ascertained. Clearly, future studies would benefit from a more comprehensive questionnaire.

The proportion of persons living with HIV who have CD4 values below 350 cells/mm^3^ is most concerning. The cause of undertreatement is not fully understood. Anecdotally, several possible reasons have been noted and a more formal study is planned. The hypothesized reasons include the change in guidelines that moved treatment eligibility from ≤ 200 to ≤ 350 cells/mm^3^, thus some patients have recently started ART and have not had time to respond. Anecdotally, other patients state that fear of ART side effects is the reason for refusing treatment. While pill counts suggest good adherence, these may overestimate actual adherence. The average daily pill burden, duration of ART regimen and treatment naïve/experienced status was not documented. Some structural barriers may have recently impacted treatment, most notably the transfer of the treatment funding between agencies that resulted in a temporary gap in treatment for some HIV+ individuals that has since been solved.

This study relies on self-reported depression symptoms through a self-administered PHQ-9 scale. The stigma surrounding mental health would suggest our data is an underestimate of the prevalence of depression in the HIV-positive population covered by the AIDS treatment center. Our estimate is lower than that seen among HIV-positive patients in high-income countries; however, it is in the range of estimates in low- and middle-income countries [12, 74–81]. We also found that self-reported depression was generally more prevalent among women than men, consistent with other published statistics. It is most likely that the estimates underestimate depression in men more substantially [36].

Self-reported mode of transmission also provides an opportunity for misclassification due to underreporting, particularly for risk behaviors that are highly stigmatized in the region (e.g., MSM, PWID) and unknown risks (e.g., nosocomial exposure). It is possible that HCV coinfection had a stronger association with depression than PWID because of underreporting of injecting drug use, the risk for HCV among PWID is greater for those who are depressed, [82] or that HCV infection is associated with an increased risk of depression [83, 84].

## Conclusions

This cross-sectional study demonstrated the presence of untreated depressive symptoms in patients receiving care at an HIV/AIDS Centre. HIV treatment might be optimized by more proactively identifying and managing depression [85, 86]. The cultural and structural barriers to coordinated mental health care may be overcome by broadening the reach of mental health services from the specialized psychiatry clinics to primary care and AIDS Centres. A prospective study would be well suited to confirm the benefits of treating depression relative to improving ART adherence and clinical outcomes among patients with HIV in moderate income countries. Such studies are needed to support any recommendations for transforming health care systems.

## Supporting information

S1 Table Point-Biserial correlation matrix of the predictor variables. (DOCX)

S2 Table Relationship between missing information and depressive symptoms. (DOCX)
