# Supplementary material for: Untreated depression among persons living with human immunodeficiency virus in Kazakhstan: A cross-sectional study
Source: PLoS One. 2018 Mar 28;13(3):e0193976. doi: 10.1371/journal.pone.0193976 (PMC5873996; doi:10.1371/journal.pone.0193976)
Supplement: S1 Table — (DOCX) [file pone.0193976.s001.docx]

**Supporting information**

**S1 Table. Point-Biserial Correlation Matrix of the Predictor Variables**

|  | Age | HIV years | ART | CD4 | HCV | PWID | Sex | Adherence | Marital Status | Education | Ethnicity | Health |
| --- | --- | --- | --- | --- | --- | --- | --- | --- | --- | --- | --- | --- |
| Age |  |  |  |  |  |  |  |  |  |  |  |  |
| HIV years | .057 |  |  |  |  |  |  |  |  |  |  |  |
| ART | .152** | .170** |  |  |  |  |  |  |  |  |  |  |
| CD4 | .050 | .056 | .234** |  |  |  |  |  |  |  |  |  |
| HCV | .081 | .227** | .077 | -.005 |  |  |  |  |  |  |  |  |
| PWID | .103* | .323** | .078 | .057 | .440** |  |  |  |  |  |  |  |
| Sex | -.165** | -.145** | -.097* | -.149* | -.207** | -.397** |  |  |  |  |  |  |
| Adherence | .080 | .096 | .077 | -.160* | -.092 | .013 | .022 |  |  |  |  |  |
| Marital status | .014 | -.022 | .106* | .027 | -.142** | -.135** | .017 | .006 |  |  |  |  |
| Education | -.007 | .007 | -.009 | .022 | .085* | .139** | -0.28 | -0.76 | -.044 |  |  |  |
| Ethnicity | .086* | .014 | -.032 | -.086* | .144** | .121** | -.003 | .001 | -.156** | .182** |  |  |
| Health | .057 | .123** | .054 | .121** | .111** | .111* | -.003 | -.118 | -.119** | -.086* | -.022 |  |

Age and HIV years are continuous.

ART: 1 = Yes, 0 = No.

CD4: 1 = “<= 350 cells/mm^3^”, 0 = “> 350 cells/mm^3^”.

HCV, PWID: 1 = Yes, 0 = No.

Sex: 1 = male, 2=female.

Marital status: 1=married/cohabitate, 0=single/previously married

Adherence: 0 = “< 90%”, 1 = “>=90%”.

College: 0 = college, 1 = secondary school and below.

Ethnicity: 1=Russian, 0 = Other.

Health: 1 = neither good nor bad and below, 0 = good/very good.

*correlation is significant at the 0.05 level (two-tailed)

**correlation is significant at 0.01 level (two-tailed)
