# Supplementary material for: Untreated depression among persons living with human immunodeficiency virus in Kazakhstan: A cross-sectional study
Source: PLoS One. 2018 Mar 28;13(3):e0193976. doi: 10.1371/journal.pone.0193976 (PMC5873996; doi:10.1371/journal.pone.0193976)
Supplement: S2 Table — (DOCX) [file pone.0193976.s002.docx]

**Supporting information**

**S2 Table. Relationship Between Missing Information and Depressive Symptoms**

| Variable with missing values | Depressive disorder = Yes | Depressive disorder = No | p-value |
| --- | --- | --- | --- |
|  | N (%) | N (%) |  |
| ID use history (n missing =40) | 5/56 (8.9%) | 35/508 (6.9%) | .364 |
| CD4 cells (n missing = 19) | 3/56 (5.4%) | 16/508 (3.1%) | .29 |
| Adherence (n missing = 165) | 19/43 (44.2%) | 146/290 (50.3%) | .451 |

Other variables with missing values included: ethnicity (n=10), age (n=9), education (n=8), marital status (n=3), and self-assessed health (n=11).
